# Effects of non-invasive brain stimulation combined with exercise training on pain and physical function in knee osteoarthritis: a systematic review and meta-analysis

**DOI:** 10.3389/fnhum.2026.1902298

**Published:** 2026-10-28

**Authors:** Min Liu, Yujing Lai, Peng Chen, Zhiyong Song, Wenxin Ma, Jia Yu, Bo Chen, Zhaoxiang Meng

**Affiliations:** 1 College of Health-Preservation and Wellness, Dalian Medical University, Dalian, Liaoning, China; 2 Department of Rehabilitation Medicine, Northern Jiangsu People’s Hospital, Yangzhou, Jiangsu, China; 3 Clinical Medical College, Yangzhou University, Yangzhou, Jiangsu, China; 4 Rehabilitation Medicine and Physiotherapy, The Yangzhou Clinical Medical College of Xuzhou Medical University, Yangzhou, Jiangsu, China; 5 Department of Rehabilitation Medicine, Yangzhou Second People’s Hospital, Yangzhou, Jiangsu, China

**Keywords:** exercise training, knee osteoarthritis, noninvasive brain stimulation, pain, physical function

## Abstract

**Background:**

Non-invasive brain stimulation (NIBS) combined with exercise training (ET) is a promising treatment for knee osteoarthritis (KOA), but its effects on pain and physical function lack systematic evaluation.

**Purpose:**

To evaluate the effects of NIBS combined with ET on pain and physical function in KOA patients.

**Methods:**

PubMed, Embase, the Cochrane Library, and Web of Science were systematically searched from inception to December 9, 2025, for randomized controlled trials (RCTs) investigating the efficacy of NIBS + ET for KOA. Primary outcomes were pain and physical function. Secondary outcomes included gait speed and quality of life (QoL).

**Results:**

This review included 8 studies with 340 participants. Our findings revealed that compared with the control, NIBS + ET showed a favorable but non-significant effect on pain measured by the numerical rating scale (NRS) or visual analog scale (VAS) (standardized mean difference [SMD] = −0.41, 95% confidence interval [CI] [−0.83, 0.01], *p* = 0.0566; I2 = 67.3%). Subgroup analyses by assessment time point, stimulation site, and NIBS modality showed no statistically significant effects. Additionally, the combined intervention significantly reduced the WOMAC total score (MD = −6.76, 95% CI [−11.85, −1.68], *p* = 0.0091; I^2^ = 51.3%). Subgroup analyses showed beneficial effects at ≥ 12 weeks (MD = −11.44, 95% CI [−15.32, −7.55], I^2^ = 0%) and for rTMS + ET (MD = −10.70, 95% CI [−16.46, −4.93], I^2^ = 19.5%). However, after accounting for within-study correlations across WOMAC outcomes, three-level meta-analyses and robust variance estimation (RVE) analyses provided weaker statistical support, with statistical significance less consistently observed across analyses. Available evidence suggested a beneficial effect on gait speed. No clear QoL improvement was observed.

**Conclusion:**

NIBS + ET may not significantly reduce NRS/VAS pain scores but was associated with reduced WOMAC total scores, although analyses accounting for within-study correlations provided weaker statistical support for WOMAC outcomes. Limited evidence suggested benefits for gait speed, whereas effects on QoL remained unclear. The certainty of evidence was moderate for physical function and low for pain, gait speed, and QoL. Further high-quality RCTs are needed to clarify these effects and optimize NIBS and ET protocols.

**Systematic review registration:**

https://www.crd.york.ac.uk/PROSPERO/view/CRD420251186212, identifier PROSPERO (CRD420251186212).

## 1 Introduction

Osteoarthritis is a common disabling illness whose global health burden continues to escalate with population aging and rising obesity rates, profoundly impacting patients’ quality of life (QoL), healthcare systems, and socioeconomic aspects (Hunter and Bierma-Zeinstra, 2019). The knee joint is the most frequently affected site in osteoarthritis. Knee osteoarthritis (KOA) is a degenerative joint disease clinically characterized by chronic pain and progressive loss of joint function, often manifesting as joint pain, stiffness, swelling, and limited mobility (Cross et al., 2014). Epidemiological data indicate that approximately 595 million people worldwide suffered from osteoarthritis in 2020, with a prevalence rate of 14.8% among individuals aged 30 and above (Kloppenburg et al., 2025). The pain and functional limitations caused by KOA not only significantly impair patients’ lower limb mobility but also impose a persistent and heavy burden on their overall QoL and physical and mental wellness (Wojcieszek et al., 2022).

Current clinical management of osteoarthritis encompasses medication, surgery, exercise, etc. However, long-term use of nonsteroidal anti-inflammatory drugs (NSAIDs) may increase the risk of cardiovascular events (da Costa et al., 2017), while surgical treatment is associated with short- and long-term complications (Matharu et al., 2020). Although exercise training (ET) is a safe and recommended approach, patients usually have difficulty maintaining regular exercise, resulting in a lack of long-term benefits (Karaborklu Argut et al., 2024). Therefore, it is imperative to explore more effective, synergistic management strategies for KOA. Early research has suggested that the pain in KOA primarily stems from injury to peripheral afferent nerves within the joint (Rakel et al., 2015). However, recent studies have revealed that the intensity of pain experienced by patients does not always correlate with the degree of their radiographic structural changes (Culvenor et al., 2019; Ohashi et al., 2023). This discrepancy suggests that, beyond peripheral mechanisms, the central nervous system (CNS) may play a pivotal role in the generation and persistence of pain (Nijs et al., 2021).

In this context, non-invasive brain stimulation (NIBS) techniques have garnered increasing attention. Among them, transcranial direct current stimulation (tDCS) and repetitive transcranial magnetic stimulation (rTMS) are the most widely applied in osteoarthritis. tDCS modulates cortical excitability by applying subthreshold currents via electrodes (Stagg and Nitsche, 2011), while rTMS alters cortical excitability using magnetic pulses generated by coils (Ziemann et al., 2008). The chronic abnormal pain and proprioceptive input in KOA patients may alter the excitability of spinal and supraspinal neurons, further inhibiting the function of the motor cortex (Mansfield et al., 2022). A systematic review and meta-analysis on chronic pain has indicated that patients with chronic pain may exhibit structural, organizational, and functional alterations in the primary motor cortex (M1), although the evidence for most neurophysiological indicators remains inconsistent (Chang et al., 2018). Despite these inconsistent findings, NIBS has demonstrated analgesic effects in chronic pain conditions such as osteoarthritis. A randomized controlled trial in older adults with KOA found that M1-targeted tDCS reduced pain scores and improved descending pain inhibitory function, as reflected by conditioned pain modulation (CPM) (Tavares et al., 2021). Meanwhile, in studies on fibromyalgia, rTMS has shown potential for alleviating pain symptoms, which may be associated with modulation of pain-processing brain regions and neurotransmitter function (Ansari et al., 2021). Beyond M1, the dorsolateral prefrontal cortex (DLPFC) is also a non-motor rTMS target for pain modulation. Evidence from Che et al. (2021) and an updated meta-analysis by Zhou et al. (2024) suggests that high-frequency DLPFC-rTMS may exert analgesic effects in chronic pain, including provoked pain, although the magnitude of benefit may vary across pain types.

Beyond neuromodulation interventions, organizations including the Osteoarthritis Research Society International (OARSI) and the American College of Rheumatology (ACR) have designated ET as the gold standard for KOA management (Bannuru et al., 2019; Kolasinski et al., 2020). The therapeutic mechanisms of ET involve enhancing cortico-thalamic excitability (Neva et al., 2017) and modulating corticospinal excitability (Pimenta et al., 2024). ET also activates dopamine pathways and the endogenous opioid system and influences neural processes related to motivation and reward, thereby modulating pain perception (Wakaizumi et al., 2016). A meta-analysis has suggested that exercise improves the muscle mass of lower limbs, neural control of movement, and range of motion of joints in individuals with KOA. These changes promote pain relief, enhance physical function, and elevate QoL (Yan et al., 2025). However, another systematic review has noted that while exercise may reduce the intensity of pain and improve physical function in chronic pain conditions like KOA, the quality of existing evidence remains suboptimal. Further high-quality studies are warranted to clarify the efficacy of ET (Geneen et al., 2017).

Based on these mechanisms, the combined application of NIBS and ET theoretically exerts a synergistic effect, but existing studies have reached inconsistent conclusions. Regarding standalone applications, some studies have indicated that tDCS and rTMS relieve chronic pain (Lloyd et al., 2020; Ciampi de Andrade and Valiengo, 2025), though their analgesic effects are influenced by stimulation parameters and subtypes of pain. They also exhibit limited efficacy in functional improvements (Li et al., 2021). Physical exercise also demonstrates short-term efficacy in relieving pain, improving function, and enhancing QoL, which is supported by low-to-moderate evidence (Lawford et al., 2024). A meta-analysis on chronic pain has suggested that the combined intervention may alleviate symptoms moderately or substantially (Cardenas-Rojas et al., 2020). A randomized controlled trial (RCT) has also indicated that rTMS combined with hip abduction training outperforms oral celecoxib in enhancing muscle strength and alleviating pain in individuals suffering from KOA (Cheng et al., 2024). Nonetheless, the findings of current clinical studies on NIBS combined with ET for KOA remain inconsistent. There is a lack of systematic reviews and meta-analyses to comprehensively assess its precise efficacy. Therefore, this study sought to integrate existing evidence from RCTs to clarify whether NIBS combined with ET more effectively alleviates pain and improves physical function in KOA patients than control, and whether such benefits can be sustained for a long term. Besides, this study also investigated differences in the efficacy of NIBS combined with ET across different geographic regions and stimulation sites.

## 2 Methods

This study was conducted following the Preferred Reporting Items for Systematic Reviews and Meta-Analyses (PRISMA) guidelines (Page et al., 2020). The study protocol was prospectively registered in the International Prospective Register of Systematic Reviews (PROSPERO) (ID: CRD420251186212) on November 12, 2025, before the initiation of the literature search.

### 2.1 Data source and search strategy

Two reviewers (ML and YJL) independently searched PubMed, Embase, the Cochrane Library, and Web of Science for articles published up to December 9, 2025. The search strategy was designed by combining Medical Subject Headings (MeSH) terms and free-text words via Boolean logic operators. The search terms included “knee osteoarthritis,” “noninvasive brain stimulation,” “transcranial magnetic stimulation,” “transcranial direct current stimulation,” and “exercise.” To avoid omissions, the reference lists of included studies were also searched to identify additional studies not covered in our initial search. Any disagreements between reviewers were resolved through discussion or consultation with a third reviewer (BC). Detailed search strategies for the databases are provided in Supplementary Table S1.

### 2.2 Eligibility criteria

The Population, Intervention, Comparison, Outcomes and Study (PICOS) framework was utilized to design the inclusion criteria of our study as follows: (i) Population: patients diagnosed with KOA according to guidelines, including the ACR guidelines, the OARSI guidelines, etc.; (ii) Intervention: NIBS, including tDCS or rTMS, combined with any type of exercise, including aerobic exercise, non-aerobic exercise, resistance exercise, or dynamic strength training; (iii) Comparison: sham NIBS combined with any type of exercise; (iv) Outcome: pain, physical function, gait speed, and QoL; (v) Study design: RCT only; (vi) Language: only studies published in English were included.

Furthermore, given the variability in participant selection criteria and study design across clinical trials, no uniform restrictions were imposed on specific eligibility criteria such as pain severity, imaging severity, or the degree of functional limitation. Differences in these inclusion criteria were not considered grounds for study exclusion, provided that participants met the predefined diagnostic criteria for KOA and the studies fulfilled the prespecified PICOS framework.

The following publications were excluded: (i) studies in which knee symptoms were primarily attributable to conditions other than KOA, including secondary knee pain, inflammatory joint diseases, or other disorders affecting knee function. (ii) studies involving participants who were unsuitable for NIBS or unable to complete the prescribed ET; (iii) duplicate publications, studies with unavailable full texts, missing data, or serious errors; (iv) clinical trial registration records; (v) animal studies; (vi) review articles such as systematic reviews and meta-analyses; (vii) other types of studies, including conference abstracts, letters, and guidelines.

### 2.3 Study selection

All retrieved records were loaded into EndNote 21. After removing duplicates, two authors (ML and YJL) independently screened all studies by reviewing titles and abstracts to identify potentially eligible studies. A full-text evaluation of potentially relevant articles was then conducted based on the predefined eligibility criteria. Any discrepancies between reviewers were resolved through discussion or consultation with a third reviewer (BC).

### 2.4 Data extraction

To avoid bias arising from data extraction by a single researcher, two independent researchers (ML and YJL) undertook study selection and data extraction. When consensus could not be reached between the two authors, a third researcher (BC) joined in the discussion and resolved discrepancies. Upon confirmation of eligible studies, the following data were extracted from each selected study into an Excel spreadsheet: (i) first author and publication year; (ii) country; (iii) sample sizes of intervention and control groups; (iv) age and sex of participants, (v) severity of disease (Kellgren-Lawrence [KL] grading, pain score), (vi) names and specific interventions or controls of intervention groups and control groups, (vii) the exercise type, stimulation modality/site/intensity, stimulation protocol/duration, intervention duration, and implementation sequence in combined interventions, (viii) Outcome measures.

### 2.5 Risk of bias and certainty of evidence assessment

Two researchers (ML and YJL) independently assessed the risk of bias of the included studies using the Cochrane Risk of Bias 2 (RoB 2) tool (Sterne et al., 2019) and evaluated the certainty of evidence for each outcome using the Grading of Recommendations Assessment, Development and Evaluation (GRADE) framework (Balshem et al., 2011). The risk of bias was classified as “low risk,” “some concerns,” or “high risk,” and the certainty of evidence was categorized as “high,” “moderate,” “low,” or “very low.” When disagreements arose between the two researchers, a third researcher (BC) was consulted to resolve discrepancies.

### 2.6 Statistical analysis

For continuous variables, effect sizes from individual studies were pooled utilizing the mean difference (MD) or standardized mean difference (SMD) with 95% confidence interval (CI). Given the heterogeneity across included studies, random-effects models were used as the primary approach for meta-analyses, regardless of the observed *I^2^* value. Heterogeneity was quantified using *I^2^* and further explored through subgroup analyses where data permitted. Additional three-level meta-analyses with robust variance estimation (RVE) were performed for outcomes for which the same study contributed multiple dependent effect sizes (Hedges et al., 2010; Cheung, 2014). For visual analog scale (VAS)/numerical rating scale (NRS) pain scores, these analyses were applied to subgroup analyses by assessment time point and stimulation site, where some studies contributed multiple dependent effect sizes. For WOMAC outcomes, three-level models with RVE were used to account for correlations among WOMAC subscales and were also applied to analyses incorporating WOMAC total scores and data from multiple assessment time points, with NIBS modality additionally examined as a moderator. For outcomes where multiple eligible intervention arms were combined before effect-size calculation or where each study contributed a single effect size, no additional multilevel analyses were performed, and conventional meta-analytic models were used. The robustness of the results was further examined across assumed within-study correlations (ρ = 0.5, 0.7, and 0.9). Additionally, a sensitivity analysis was performed using the leave-one-out approach (Patsopoulos et al., 2008) to determine whether any single study substantially influenced the overall results. Potential publication bias was assessed by analyzing funnel plots (Egger et al., 1997) and further explored using the trim-and-fill method. For all outcome variables, *p* < 0.05 was considered statistically significant. All statistical analyses were performed with R v4.5.2.

## 3 Results

### 3.1 Literature selection results

A total of 182 studies were retrieved from 4 databases. Using EndNote 21 software, 64 duplicate records were removed, leaving 118 studies for screening. Of the 14 studies passing the initial screening, 3 were not retrieved, and the remaining 11 underwent full-text review. During the full-text review, 3 studies were excluded: one due to an ineligible intervention, one due to an ineligible control, and one due to an ineligible study design. Ultimately, 8 studies were included. Figure 1 displays the literature retrieval and assessment flowchart.

**Figure 1:**
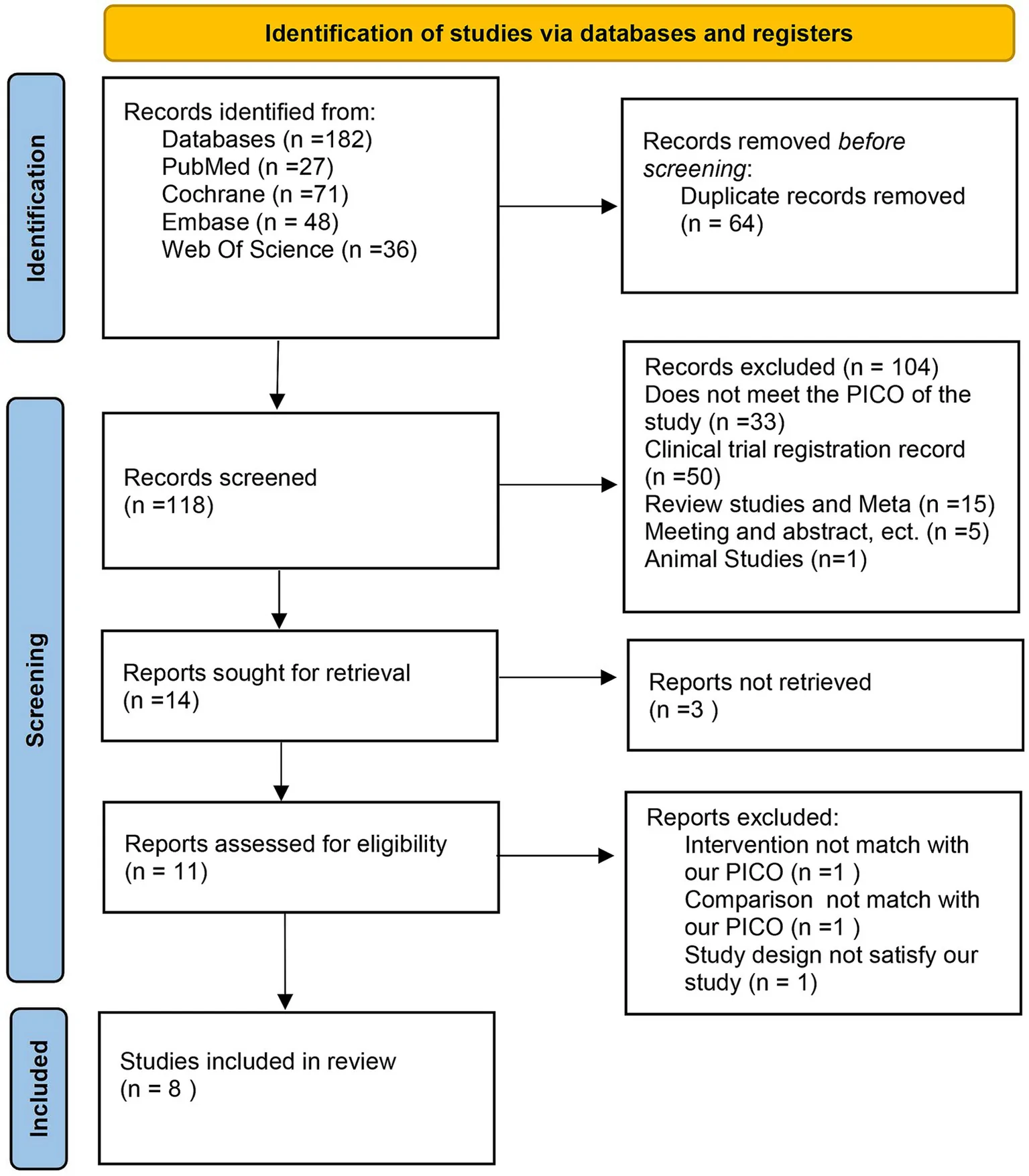
Literature retrieval and assessment flowchart.

### 3.2 Study characteristics

Table 1 details the characteristics of the 8 studies (Wang et al., 2025; Kalkhoran and Khanmohammadi, 2025; Kaboodi et al., 2025; Chang et al., 2025; Boonprasit et al., 2025; Montilla-Herrador et al., 2024; Chang et al., 2017; Rahimi et al., 2021) included in our analysis. These studies involved 340 patients with KOA across Asian and non-Asian regions, including China, Iran, Thailand, Australia, and Spain. Sample sizes ranged from 30 to 80. The mean age of participants varied from 54 to 70 years. Two studies enrolled only women, while the remaining six included both male and female patients. Regarding disease severity, six studies utilized K-L grading as an inclusion criterion, covering grades 1 to 4, while two studies had no restrictions on grading. Seven studies specified requirements for baseline pain scores, while one study did not. For outcome measures, pain was assessed in seven studies, three utilizing the VAS (0–10 points), two using the VAS (0–100 points), and two using the NRS (0–10 points). For physical function assessment, six studies adopted the Western Ontario and McMaster Universities Osteoarthritis Index (WOMAC), while one study utilized the Knee Injury and Osteoarthritis Outcome Score (KOOS). Additionally, three studies reported 10-meter walking speed, while three studies assessed QoL utilizing the Osteoarthritis Knee and Hip Quality of Life (OAKHQOL) questionnaire, the European Quality of Life 5 Dimensions (EQ-5D) scale, and the Short Form Health Survey-36 (SF-36), respectively. The baseline characteristics for the included studies are summarized in Table 1, with detailed diagnostic criteria for KOA and the inclusion and exclusion criteria for each study presented in Supplementary Table S2.

**Table 1:** Characteristics of studies included in this review.

References	Country	Sample size (I/C)	Age (I, Mean ± SD)	Age (C, Mean ± SD)	Sex (I, M/F)	Sex (C, M/F)	KL grading	Pain score	Intervention (I)	Control (C)	Outcomes
Wang et al. (2025)	China	24/24	63.63 ± 5.347	64.04 ± 4.319	5/19	6/18	II ~ III	VAS ≤ 5/10	High-frequency rTMS + Quadriceps training + Health education	Sham rTMS + Quadriceps training + Health education	(0–10) VAS, WOMAC
Kalkhoran and Khanmohammadi (2025)	Iran	17/17	65.47 ± 1.62	65.05 ± 1.34	Female (17)	Female (17)	≥I	\	Real tDCS + Aquatic Therapy	Sham tDCS + Aquatic Therapy	10-Meter Walk Test
Kaboodi et al. (2025)	Iran	20/20	55.90 ± 9.85	54.80 ± 7.22	Female (20)	Female (20)	≥II	VAS ≤ 4/10	Real tDCS + Aquatic neuromuscular training	Sham tDCS + Aquatic neuromuscular training	(0–10) NRS, WOMAC, Osteoarthritis knee and hip quality of life
Chang et al. (2025)	Australia	17/14	64.2 ± 7.6	67.1 ± 9.6	5/12	5/9	\	NRS ≥ 4/10	High-frequency rTMS + Strengthening exercises	Sham rTMS + Strengthening exercises	(0–10) NRS, WOMAC
Boonprasit et al. (2025)	Thailand	16/16	64.44 ± 5.56	65.50 ± 7.06	1/15	2/14	I ~ III	VAS = (40–70)/100	Real tDCS + Resistance exercise + Education	Sham tDCS + Resistance exercise + Education	(0–100) VAS, WOMAC, SF-36
Montilla-Herrador et al. (2024)	Spain	24/21	64.96 ± 7.20	70.95 ± 8.26	5/19	7/14	I ~ IV	VAS ≥ 4/10	Real tDCS + Sham TENS + Neuromuscular exercise + Education	Sham tDCS + Sham TENS + Neuromuscular exercise + Education	(0–10) VAS, WOMAC, 10-Meter Walk Test, EQOL-5D
Chang et al. (2017)	Australia	15/15	59.8 ± 9.1	64.1 ± 11.1	4/11	6/9	\	VAS ≥ 40/100	Real tDCS + Strengthening exercises	Sham tDCS + Strengthening exercises	(0–100) VAS, WOMAC
Rahimi et al. (2021)	Iran	20(M1)/20(DLPFC)/20(S1)/20	58.8 ± 3.3	58.8 ± 3.3	90% of the individuals were female		II ~ III	VAS ≥ 4/10	M1/DLPFC/S1 tDCS + Physical therapy	Sham tDCS + Physical therapy	(0–10) VAS, KOOS, 10-Meter Walk Test

I = Intervention group; C = Control group; rTMS = repetitive transcranial magnetic stimulation; tDCS = transcranial direct current stimulation; TENS = transcutaneous electrical nerve stimulation; M1 = primary motor cortex; DLPFC = dorsolateral prefrontal cortex; S1 = primary somatosensory cortex; VAS = visual analogue scale; NRS = numeric rating scale; WOMAC = Western Ontario and McMaster Universities Osteoarthritis Index; KOOS = Knee injury and Osteoarthritis Outcome Score; SF-36 = Short Form Health Survey-36; EQOL = European Quality of Life Scale; SD = Standard Deviation.

### 3.3 Characteristics of combined interventions

The specific parameters of the combined interventions are detailed in Table 2. Among the eight studies, six employed tDCS and two utilized rTMS. In the studies on tDCS, the primary targets of anodal stimulation were the M1 and the DLPFC. Additionally, one study separately targeted M1, DLPFC, and the primary somatosensory cortex (S1) in different intervention groups, while another study used a dual-channel device to simultaneously stimulate M1 and the right posterior parietal cortex (PPC). In most studies, the cathode was placed in the supraorbital region (SO), while in one study, the cathode was placed at the frontal pole (Fp1, Fp2). Most studies applied stimulation at an intensity of 2 mA or 1 mA, while only one study applied stimulation at 1.5 mA. A single stimulation session lasted mostly 20 min, except for one study, which applied 30-min sessions. For sham stimulation, most tDCS studies employed electrodes at identical locations but applied only brief currents to simulate the sensation of real stimulation. Regarding the sequence of interventions, training was implemented after tDCS in four studies and simultaneously with tDCS in one study, while the remaining one study did not specify the sequence.

**Table 2:** General information on combined interventions included in the meta-analysis.

Reference	Exercise type	Stimulation modality	Sham stimulation details	Anodal/real stimulation site	Cathodal/sham stimulation site	Intensity	Stimulation protocol/duration (minutes)	Frequency (per week)	Total period (wk)	Sequence of application
Wang et al. (2025)	Quadriceps isokinetic strength training (Biodex dynamometer)	rTMS	Coil perpendicular to head, rotated 90° sagittally (simulated noise)	Contralateral M1 (lower limb motor hotspot)	\	80% rMT	1,400 pulses (10 Hz, 20 trains×7 s, ITI 55 s)	5	12	\
Kalkhoran and Khanmohammadi (2025)	Aquatic therapy	tDCS	Mimics initial sensation, no active current	M1 (C3) and right PPC (P4)	Fp1, Fp2	2 mA	30	2	8	Real tDCS before aquatic sessions
Kaboodi et al. (2025)	Aquatic neuromuscular training	tDCS	Current ramped up over 30s to mimic sensation then returned to baseline	M1 (C3 or C4) contralateral to the most affected knee	Ipsilateral SO	2 mA	20	3	6	Training immediately after tDCS
Chang et al. (2025)	Standardized quadriceps strengthening exercises	rTMS	Sham coil (no magnetic pulse, simulated clicks)	Contralateral M1 (motor hotspot for the hand)	\	90%rMT	3,000 stimuli (10 Hz, 30 trains×10s, ITI 20s)	2	6	rTMS immediately before supervised strengthening exercise
Boonprasit et al. (2025)	Progressive resistance open-kinetic chain isotonic quadriceps strengthening exercise	tDCS	30s current delivery then gradual ramp-down	Left DLPFC (F3)	Right SO	2 mA	20	3	4	Training during tDCS (after 10 min current)
Montilla-Herrador et al. (2024)	Neuromuscular exercise	tDCS	15 s ramp-up to 1.5 mA then off	Contralateral M1	Ipsilateral SO	1.5 mA	20	5	2	\
Chang et al. (2017)	Standardized quadriceps strengthening exercises	tDCS	15 s on (simulated itching) then off	Contralateral M1	Ipsilateral SO	1 mA	20	2	8	tDCS immediately before strengthening
Rahimi et al. (2021)	Physiotherapy-related exercise therapy	tDCS	30s ramp-up to 1 mA then off	Left M1/DLPFC/S1	Right SO	1 mA	20	5	2	tDCS before physiotherapy

tDCS = Transcranial Direct Current Stimulation; rTMS = repetitive Transcranial Magnetic Stimulation; rMT = resting motor threshold; M1 = Primary motor cortex; PPC = Posterior parietal cortex; Fp1 = Frontopolar region 1; Fp2 = Frontopolar region 2; SO = Supraorbital; DLPFC = Dorsolateral prefrontal cortex; S1 = primary somatosensory cortex; ITI = Intertrain interval.

In two rTMS studies, the stimulation was targeted at M1 and applied to lower limb and hand motor hotspots, respectively. The stimulation frequency was 10 Hz for both, with pulse counts of 1,400 and 3,000, and intensities of 80 and 90% of resting motor threshold, respectively. For sham stimulation, in one study, the coil edge was positioned perpendicular to the head and rotated 90° in the sagittal plane to simulate sound. In the other study, a sham coil with an identical appearance was used, which emitted only sound without generating magnetic pulses. Regarding the sequence of interventions, intensive exercise was implemented immediately after stimulation in one study, while the other study did not specify the sequence.

Among all eight studies, five studies involved strength training, primarily quadriceps strength training, including standardized exercise, progressive resistance open kinetic chain exercise, and comprehensive training integrating neuromuscular exercise. Two studies employed hydrotherapy, specifically aquatic therapy and aquatic neuromuscular training. Additionally, one study utilized physiotherapy-related exercise therapy.

### 3.4 Risk of bias assessment results

The risk of bias of the included studies was assessed using the RoB 2 tool, with the results presented in Supplementary Figure S1. Overall, five studies were judged as having a low risk of bias, two raised some concerns, and one had a high risk of bias. The certainty of evidence was further assessed using the GRADE approach (Supplementary Table S3). The certainty of evidence was moderate for physical function and low for pain, gait speed, and QoL. Downgrading was primarily due to inconsistency, imprecision, and risk of bias.

### 3.5 Pooled results for each outcome measure

#### 3.5.1 Primary outcome

##### 3.5.1.1 Pain score

Seven included RCTs (*n* = 289 patients) evaluated pain measured using the VAS or NRS in KOA patients. As shown in Figure 2, compared with the control group, the NIBS combined with ET group demonstrated a trend toward lower pain scores, although the difference was not statistically significant (SMD = −0.41, 95% CI [−0.83, 0.01], *p* = 0.0566), with an *I^2^* of 67.3%. A subgroup analysis was conducted by dividing data into three subgroups (≤4 weeks, 6–8 weeks, and ≥12 weeks). No statistically significant effects were observed at any assessment time point, including ≤ 4 weeks (SMD = −0.54, 95% CI [−1.38, 0.31], *I^2^* = 83.2%), 6–8 weeks (SMD = −0.49, 95% CI [−1.18, 0.20], *I^2^* = 79.2%) and ≥ 12 weeks (SMD = −0.37, 95% CI [−0.79, 0.05], *I^2^* = 0%) (Supplementary Figure S2). Subgroup analysis by stimulation site suggested that neither stimulation of the motor cortex (SMD = −0.40, 95% CI [−0.82, 0.02], *I^2^* = 57%) nor stimulation of the DLPFC (SMD = −0.88, 95% CI [−2.33, 0.58], *I^2^* = 88.1%) showed statistically significant effects on pain outcomes (Supplementary Figure S3). Only one study contributed data for S1 stimulation, precluding a meaningful pooled estimate for this subgroup. Furthermore, subgroup analysis by stimulation modality indicated that neither tDCS combined with ET nor rTMS combined with ET demonstrated statistically significant effects on relieving pain (SMD = −0.40, 95% CI [−0.98, 0.18], *I^2^* = 77.3%; SMD = −0.44, 95% CI [−0.91, 0.03], *I^2^* = 0%, respectively) (Supplementary Figure S4).

**Figure 2:**
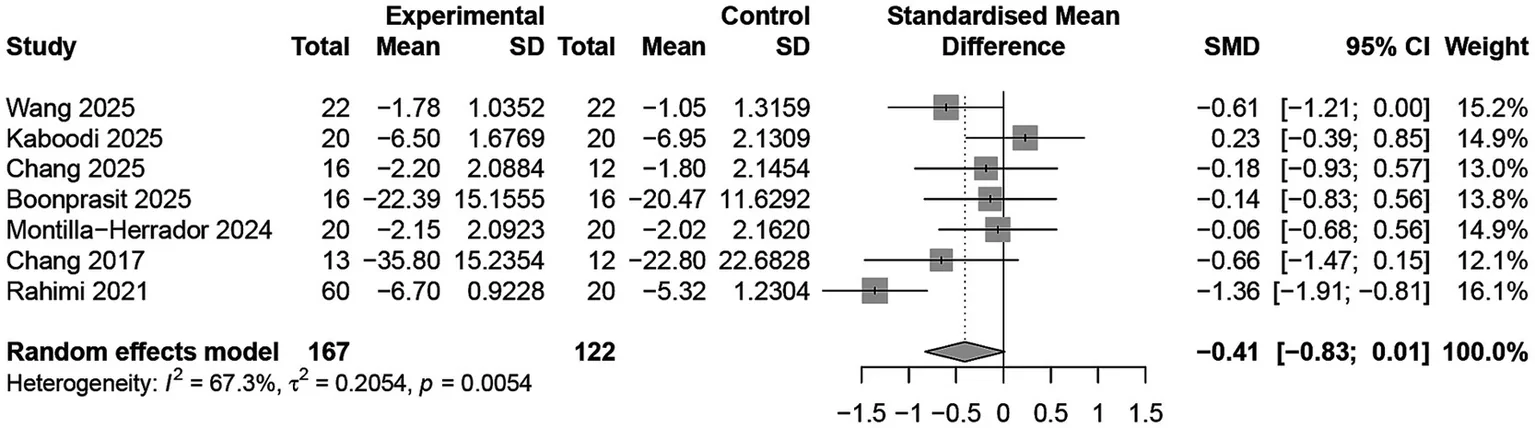
Forest plot for effects of non-invasive brain stimulation combined with exercise on pain score.

Additional three-level meta-analyses with RVE were conducted for subgroup analyses of VAS/NRS pain scores by assessment time point and stimulation site. For pain estimates across different assessment time points, the three-level model and RVE yielded results consistent with the conventional meta-analysis, showing a similarly favorable but non-significant effect (three-level model: Hedges’ *g* = −0.40, 95% CI [−0.88, 0.09], *p* = 0.098; RVE: *g* = −0.40, 95% CI [−0.93, 0.13], *p* = 0.117) (Supplementary Table S4). Similarly, the subgroup analysis by stimulation site yielded consistent estimates (three-level model: *g* = −0.39, 95% CI [−0.86, 0.07], *p* = 0.086; RVE: *g* = −0.39, 95% CI [−0.89, 0.11], *p* = 0.102) (Supplementary Table S5). Moderator analyses showed no evidence that assessment time point (*p* = 0.907) or stimulation site (*p* = 0.174) significantly influenced the effect estimates. Results remained stable across assumed within-study correlations (ρ = 0.5–0.9).

##### 3.5.1.2 Physical function score

Seven included RCTs reported physical function outcomes. Among them, six studies employed the WOMAC scale, and one utilized the KOOS scale. Therefore, only the studies employing the WOMAC scale were included in the meta-analysis, comprising 209 patients with KOA. The WOMAC total score and the scores of its three subscales, including pain (0–20), stiffness (0–8), and physical function (0–68), were analyzed separately. Higher scores indicate more severe knee joint dysfunction and symptoms of KOA. The meta-analysis revealed that NIBS combined with ET substantially reduced the WOMAC total score (MD = −6.76, 95% CI [−11.85, −1.68], *p* = 0.0091) with an *I^2^* of 51.3% (Figure 3) and the score of the pain subscale (MD = −1.18, 95% CI [−2.07, −0.29], *p* = 0.0091) with an *I^2^* of 0% (Supplementary Figure S5). However, it exhibited no significant effects on the score of the physical function subscale (MD = −4.12, 95% CI [−8.64, 0.40], *p* = 0.0740) with an *I^2^* of 58.3% (Supplementary Figure S6) or the stiffness subscale (MD = −0.61, 95% CI [−1.55, 0.34], *p* = 0.2079) with an *I^2^* of 57.1% (Supplementary Figure S7).

**Figure 3:**
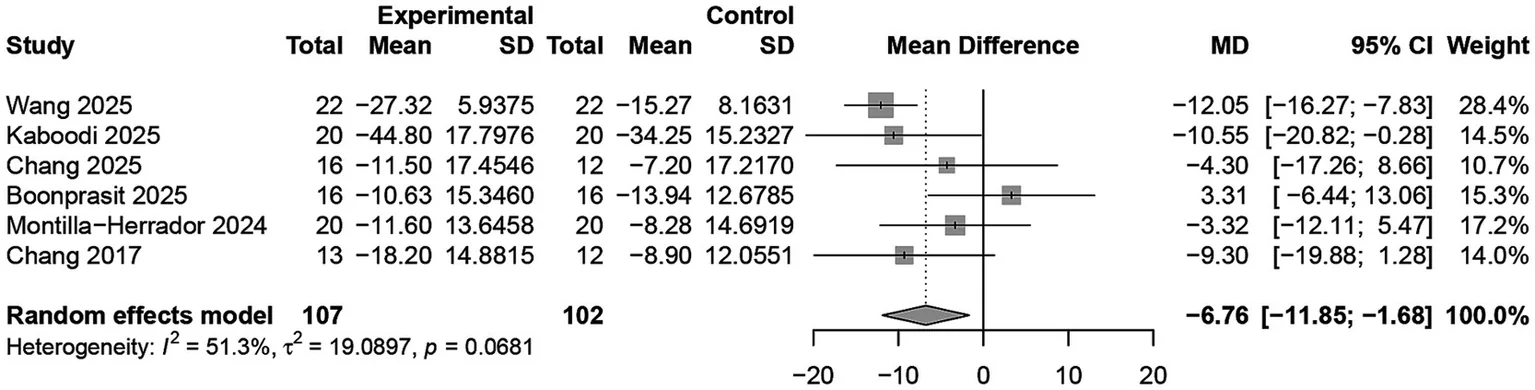
Forest plot for effects of non-invasive brain stimulation combined with exercise on WOMAC total score.

Subgroup analysis by WOMAC total score revealed that NIBS combined with ET did not significantly outperform the control at ≤4 weeks (MD = −0.35, 95% CI [−6.88, 6.18], *I^2^* = 0%) and at 6–8 weeks (MD = −5.04, 95% CI [−12.01, 1.93], *I^2^* = 36.8%), but significantly outperformed the control at ≥12 weeks (MD = −11.44, 95% CI [−15.32, −7.55], *I^2^* = 0%), as shown in Figure 4. Furthermore, subgroup analysis by stimulation modality revealed that tDCS combined with ET did not demonstrate statistically significant reductions in WOMAC total scores (MD = −4.70, 95% CI [−10.79, 1.38], *I^2^* = 35.6%). For rTMS combined with ET, a statistically significant reduction was observed (MD = −10.70, 95% CI [−16.46, −4.93], *I^2^* = 19.5%) (Supplementary Figure S8). Further subgroup analyses showed statistically significant reductions in the WOMAC pain subscale (MD = −1.68, 95% CI [−2.74, −0.63], *I^2^* = 0%) (Supplementary Figure S9) and physical function subscale (MD = −7.38, 95% CI [−12.54, −2.21], *I^2^* = 28.3%) (Supplementary Figure S10) for rTMS combined with ET. No statistically significant effects were observed for tDCS combined with ET (MD = −0.54, 95% CI [−1.89, 0.82], *I^2^* = 0.2%; MD = −1.99, 95% CI [−7.43, 3.45], *I^2^* = 41.4%, respectively).

**Figure 4:**
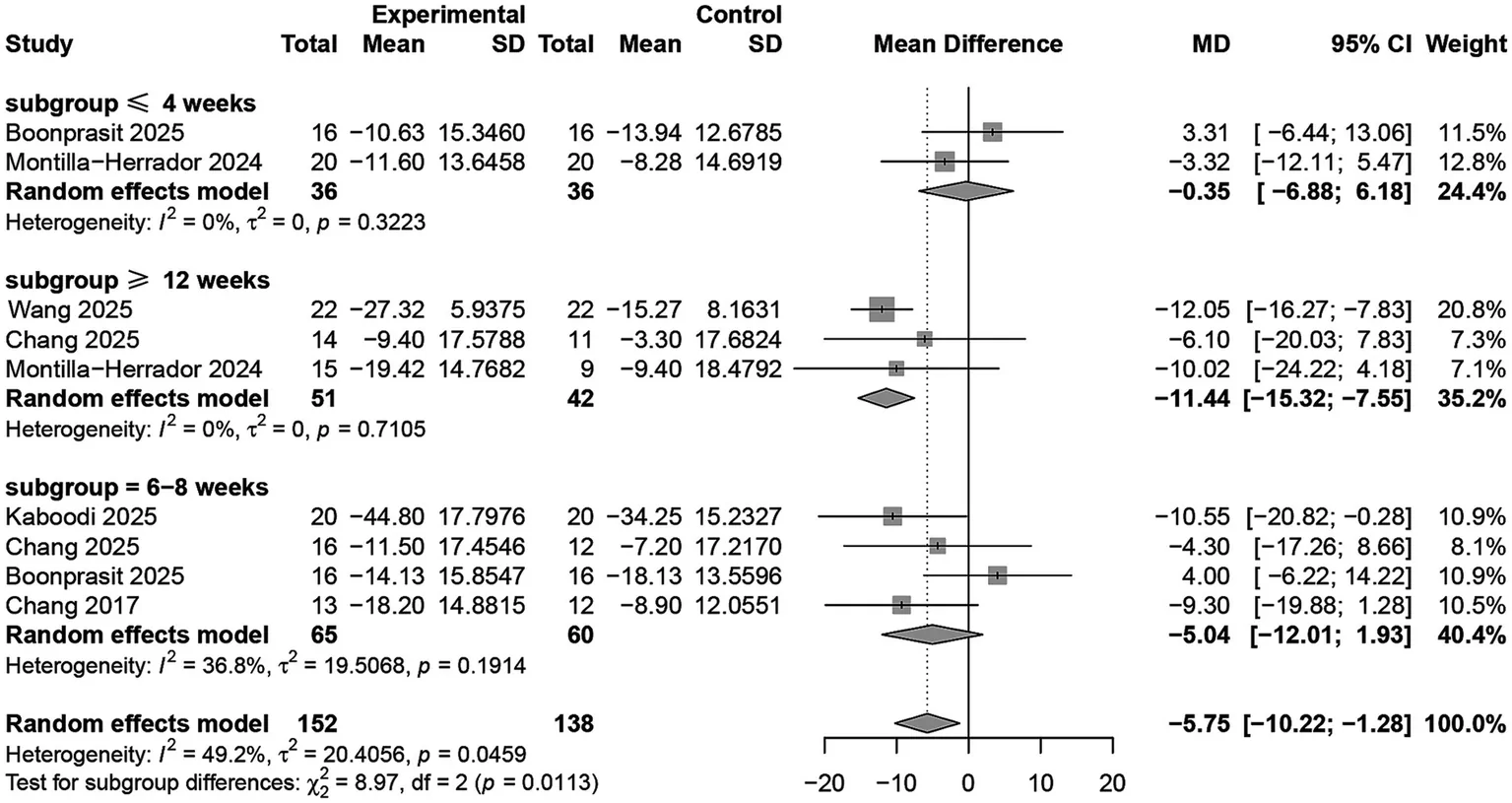
Forest plot of subgroup analysis by time for effects of non-invasive brain stimulation combined with exercise on WOMAC total score.

To account for correlated WOMAC outcomes within studies, additional three-level meta-analyses with RVE were performed (Supplementary Table S6). Across the three WOMAC subscales (13 effect sizes from 5 studies), the pooled effect was favorable but not statistically significant in either the three-level model (*g* = −0.38, 95% CI [−0.81, 0.05], *p* = 0.081) or the RVE analysis (*g* = −0.38, 95% CI [−0.94, 0.18], *p* = 0.135). Estimates remained stable across assumed within-study correlations (ρ = 0.5–0.9). Moderator analyses did not detect significant variation in treatment effects across WOMAC subscales (*p* = 0.843) or NIBS modalities (*p* = 0.119). An additional analysis incorporating the WOMAC total score (19 effect sizes from 6 studies) showed a consistent direction of effect, although the magnitude of the pooled effect appeared less pronounced (three-level model: *g* = −0.43, 95% CI [−0.82, −0.05], *p* = 0.030; RVE: *g* = −0.43, 95% CI [−0.91, 0.05], *p* = 0.068). However, after accounting for within-study correlations, the RVE-adjusted estimate was no longer statistically significant. No significant moderation by WOMAC subscale (*p* = 0.676) or NIBS modality (*p* = 0.226) was detected in this sensitivity analysis.

Additionally, for WOMAC total scores reported at multiple assessment time points, the three-level model showed a significant reduction (MD = −6.49, 95% CI [−12.65, −0.33], *p* = 0.041), whereas RVE yielded the same point estimate with a wider confidence interval, and the estimate was no longer statistically significant (MD = −6.49, 95% CI [−13.63, 0.65], *p* = 0.066) (Supplementary Table S7). Moderator analysis showed no significant effect modification by assessment time point (*p* = 0.127). Results remained generally stable across the assumed within-study correlations.

#### 3.5.2 Secondary outcomes

In three studies, gait speed was assessed by the 10-meter walk test. In another three studies, the QoL was evaluated. Regarding gait speed, a statistically significant difference was observed between the NIBS combined with ET groups and the control groups (SMD = −0.35, 95% CI [−0.69, −0.01], *p* = 0.0423) with an *I^2^* of 0% (Supplementary Figure S11). The meta-analysis of QoL showed no between-group differences (SMD = 0.20, 95% CI [−0.17, 0.57], *p* = 0.2951) with an *I^2^* of 0% (Supplementary Figure S12).

##### 3.5.2.1 Safety

Among 8 studies, 4 reported adverse effects (Wang et al., 2025; Boonprasit et al., 2025; Montilla-Herrador et al., 2024; Chang et al., 2017). Specifically, one study on rTMS mentioned scalp pain at the stimulation site during treatment, which gradually resolved after the coil was removed. One study on tDCS indicated that fatigue was the most commonly reported symptom in the active stimulation plus exercise group, while dizziness was most frequent in the sham stimulation plus exercise group. In another study on tDCS, skin redness was a common adverse effect, while other effects included tingling, itching, headache, and insomnia, with no serious adverse events reported. Still another study on tDCS showed that one participant in the intervention group experienced a headache attack after a single treatment session, while one participant in the control group reported transient pain beneath the electrode during the initial stage of stimulation. Among the remaining studies, three reported no intervention-related adverse events (Kaboodi et al., 2025; Chang et al., 2025; Rahimi et al., 2021), and one reported no adverse events (Kalkhoran and Khanmohammadi, 2025).

### 3.6 Sensitivity analysis and publication bias

Sensitivity analysis for pain scores was conducted to assess the robustness of the results (Supplementary Figure S13). The study by Rahimi et al. (2021) was identified as the primary contributor to the observed heterogeneity. Upon exclusion of this study, the pooled effect size was attenuated and remained non-significant (SMD = −0.21, 95% CI [−0.50, 0.07], *p* = 0.1401), with substantially reduced heterogeneity (*I^2^* = 0%) (Supplementary Figure S14), indicating that the overall findings were robust despite the influence of this study on the pooled estimate. Additionally, a sensitivity analysis for the WOMAC total score showed generally consistent pooled estimates, although exclusion of Wang 2025 resulted in a loss of statistical significance (Supplementary Figure S15). Given the small number of included studies, funnel plots (Supplementary Figures S16, S17) were considered exploratory, and Egger’s test was not performed. Trim-and-fill analysis imputed two studies for pain, changing the statistical significance of the pooled effect (Supplementary Figure S18), whereas three studies were imputed for the WOMAC total score, with statistical significance maintained despite an increased effect magnitude (Supplementary Figure S19).

## 4 Discussion

This systematic review and meta-analysis included 8 RCTs, aiming to evaluate the effects of NIBS (including tDCS and rTMS) combined with ET on pain and physical function in patients with KOA. The results indicated a consistent but non-significant trend toward reduced pain, as measured by the NRS or VAS, in the NIBS combined with ET groups compared with the sham stimulation combined with exercise groups. Subgroup analyses by assessment time point, stimulation site, and stimulation modality also revealed no significant therapeutic benefit. Additional analyses accounting for within-study correlations yielded similar findings, with no significant modifying effects of assessment time point or stimulation site observed. Additionally, the intervention groups showed a significant reduction in the WOMAC total score. Subgroup analyses suggested improvement at ≥12 weeks, whereas no clear evidence of improvement was observed at ≤4 weeks or 6–8 weeks. Subgroup analysis by stimulation modality further showed that rTMS combined with ET was associated with reduced WOMAC total scores. No clear improvements were observed for tDCS combined with ET. Moreover, the findings suggested improvement in the WOMAC pain subscale, whereas improvements in WOMAC physical function and stiffness subscales were not clearly demonstrated. Nevertheless, after accounting for within-study correlations, no significant differences were detected among the WOMAC subscales. NIBS modality also did not significantly modify the treatment effects on WOMAC outcomes. Analyses including either the WOMAC subscales alone or both the WOMAC total score and subscales showed a favorable direction of effect, although the RVE-adjusted estimates were not statistically significant. In addition, assessment time point did not significantly modify the effect on the WOMAC total score. Available evidence suggested a beneficial effect on gait speed. No clear improvement was observed in QoL.

Chronic pain in KOA stems from the dysregulation of the neural pathway from joint receptors to the spinal cord and brain. It is characterized by hypersensitivity of nociceptors, heightened excitability with diminished inhibitory function in dorsal horn neurons of the spinal cord, and structural and functional alterations in brain regions including the thalamus, brainstem, and cortex (Fu et al., 2018; Shi and Wu, 2023; Apkarian et al., 2005; Fornasari, 2012; Borsook, 2012). These central pain-related brain regions do not function in isolation but appear to be interconnected within cortical and subcortical networks. NIBS targeting a local cortical region can influence pain-related brain regions beyond the stimulation site through these functional connections (Kong et al., 2024).

Given the mechanisms of KOA pain and the central pain-modulatory effects of NIBS, several studies have proposed possible mechanisms underlying NIBS combined with ET. Chang et al. (2017) suggested that the pain reduction observed with tDCS combined with quadriceps strengthening exercises may be related to a priming effect of tDCS, whereby tDCS modulates central pain processing and enhances cortical responsiveness to subsequent motor training. Wang et al. (2025) noted that the pain-relieving effect of NIBS combined with ET arises from the synergy between the central and peripheral nervous systems. Specifically, high-frequency rTMS may modulate brain excitability to relieve pain and improve motor control, while ET reduces joint damage and inflammation by increasing muscle strength and improving biomechanics. Similar effects were observed in patients with fibromyalgia, where tDCS combined with aerobic exercise outperformed either intervention alone, with further improvements in pain, anxiety, and emotional outcomes (Mendonca et al., 2016). Although a statistically significant improvement in pain assessed by VAS/NRS following NIBS combined with ET in patients with KOA was not identified in this meta-analysis, the overall pooled effect size showed a favorable trend toward the combined intervention. This finding is partially consistent with the meta-analysis by Xia et al. (2025) in patients with chronic low back pain, which indicated that the additional analgesic effect of tDCS beyond ET was relatively limited. Although our findings showed a similar direction of effect, the pooled estimate was accompanied by substantial between-study heterogeneity, which persisted in several pain subgroup analyses and was not clearly explained by assessment time point, stimulation site, or stimulation modality. Teixeira et al. (2020) also highlighted that substantial clinical heterogeneity and a limited number of studies remain key factors limiting the interpretation of the effects of a combined intervention.

Notably, although no significant effect was observed for pain assessed by VAS/NRS in the present study, a significant reduction was identified in the WOMAC pain subscale. Previous studies have indicated that OA-related pain is multidimensional, involving persistent pain, intermittent pain, and activity-related pain (O'Neill and Felson, 2018; Salaffi et al., 2014). The WOMAC pain subscale primarily reflects pain experienced during physical activities rather than providing an independent global assessment of pain severity (Gandek, 2015). Therefore, these two outcome measures may capture different aspects of pain experience and may differ in their sensitivity to treatment-related changes. Moreover, multiple systematic reviews have supported ET as an effective intervention for relieving pain in patients with KOA (Yan et al., 2025; Lawford et al., 2024; Mo et al., 2023). Importantly, KOA-related pain is not solely mediated by central sensitization but also involves multiple mechanisms, including synovial inflammation, bone marrow lesions, and persistent peripheral nociceptive input (Eitner et al., 2017). Since this study evaluated the incremental benefit of NIBS added to ET, the incremental pain-relieving effect achieved through central neuromodulation may be limited by the complex pathological mechanisms underlying pain caused by KOA, making statistically significant differences more difficult to detect. Although previous meta-analyses support the favorable effect of NIBS combined with exercise for chronic pain, current evidence on the additional effect of combined interventions remains limited (Cardenas-Rojas et al., 2020). In particular, factorial-designed studies that simultaneously compare NIBS alone, ET alone, and their combination are lacking.

The combined interventions showed more consistent and pronounced improvement in the WOMAC total score despite the absence of statistically significant efficacy in pain outcomes, with favorable but non-significant effects on the physical function subscales. Improvements in physical function are not entirely dependent on pain reduction but are influenced by multiple factors, including muscle strength, proprioception, and joint stability (Øiestad et al., 2022; Knoop et al., 2011). Therefore, even in the absence of significant additional analgesic effects, the combined interventions may still improve overall physical function by promoting motor learning and enhancing the effectiveness of ET (Reis and Fritsch, 2011; Rumpf et al., 2017; Hamoudi et al., 2018). A systematic review has indicated that enhanced thigh muscle strength, reduced joint extension disorder, and improved proprioception are key mediating factors in exercise-induced alleviation of symptoms in KOA (Runhaar et al., 2015). Furthermore, ET offers comprehensive benefits for alleviating KOA by improving neuromuscular function, enhancing joint stability, regulating cartilage metabolism, reducing systemic inflammation, and promoting psychosocial well-being (Beckwée et al., 2013).

Building upon this, the combination of NIBS and ET may exert therapeutic effects through a dual pathway of central modulation and peripheral functional enhancement, as confirmed by multiple studies. Chronic abnormal joint sensory input can cause motor cortex inhibition in KOA patients, thereby reducing the efficiency of muscle activation and the effectiveness of strength training (Sherman et al., 2024). tDCS targeting M1 enhances cortical excitability, reduces motor reaction time, and improves the efficiency of motor unit recruitment, potentially modulating both pain perception and motor output (Stagg et al., 2012). Clinical research has indicated that in women with KOA, tDCS combined with aquatic neuromuscular training significantly improves functional performance, with greater improvement observed in the real stimulation group (Kaboodi et al., 2025). This improvement may be attributed to tDCS-induced modulation of M1 excitability, which optimizes motor control and synergistically enhances neuroplasticity and motor learning capability in conjunction with training. Similarly, rTMS combined with quadriceps strengthening training induces significant functional improvement only in the real stimulation group, suggesting that rTMS may exert a “priming” effect by activating specific brain regions to enhance motor signal integration and motor skill consolidation (Chang et al., 2025). Collectively, these findings indicate that NIBS combined with ET synergistically modulates the CNS and regulates peripheral adaptation (e.g., increasing the cross-sectional area of muscles, suppressing inflammation; Zeng et al., 2021), thereby resulting in more stable improvement in physical function. Notably, the combined interventions did not result in a significant reduction in the WOMAC stiffness score. This may be attributed to the complex and multifactorial pathological basis of KOA-related stiffness, which is not only associated with pain and functional impairment but may also involve inflammatory responses, joint fibrosis, and abnormal neuromuscular control (Uritani et al., 2023).

However, because the WOMAC total score and its subscale scores were derived from the same measurement system, the effect sizes within studies were not completely independent. The RVE-adjusted three-level meta-analysis found no significant differences among the WOMAC subscales, suggesting that, given the limited number of available studies, the apparent relative advantage of individual subscales may not be stable. Whether the analysis included only the three WOMAC subscales or both the WOMAC total score and WOMAC subscales, the effect remained favorable, although the magnitude of the pooled effect differed somewhat between the two analyses. In both analyses, the RVE-adjusted estimates did not reach statistical significance, which may reflect increased uncertainty after accounting for within-study correlations and the limited number of independent studies. Therefore, the beneficial effects of NIBS combined with ET on WOMAC outcomes require further validation.

Our study further revealed that the combined intervention may have sustained benefits for physical function improvement. A potential reason is that the alleviation of pain-related symptoms through the combined interventions during the early stage (Kaboodi et al., 2025) may enable patients to better participate in and complete ET, thereby initiating a virtuous cycle of functional improvement earlier. Besides, NIBS directly modulates functional connectivity within brain networks (Nie et al., 2025). Such alterations in the CNS may be more enduring than peripheral adaptation alone. Similarly, improvements in muscle strength, motor skill acquisition, and neuromuscular adaptation induced by ET generally require repeated practice and cumulative time-dependent processes (Dayan and Cohen, 2011; Kleim and Jones, 2008). Therefore, the benefits of combined interventions may become increasingly apparent at later assessment time points, reflecting the gradual development of functional adaptations over time. However, analyses accounting for within-study correlations did not demonstrate a significant modifying effect of assessment time point. Therefore, the influence of assessment time point on the treatment effect remains uncertain. Current evidence also suggests that the therapeutic effects of NIBS are influenced by stimulation parameters and patient characteristics, and its long-term efficacy remains unclear (Zhu et al., 2022). Therefore, further studies with longer follow-up periods are warranted to confirm the durability of these effects.

Subgroup analysis also showed that rTMS combined with ET was associated with improvements in the WOMAC total, as well as its pain and physical function subscale scores. rTMS may alleviate chronic pain in patients with KOA characterized by central sensitization by modulating pain regulation networks (Lluch et al., 2014; Nguyen et al., 2019). A network meta-analysis found that both 10-Hz and 20-Hz rTMS were associated with greater pain reduction than 5-Hz rTMS and control conditions (Naik et al., 2024). Both studies included in the rTMS subgroup of the present review used 10-Hz stimulation. However, given that this subgroup included only two studies, these findings should be considered exploratory, and the extent to which stimulation frequency contributed to the observed effects cannot be determined. rTMS combined with ET did not significantly improve pain assessed by VAS/NRS, despite evidence of analgesic effects of rTMS in other chronic pain populations (Abdelsalam et al., 2026). This discrepancy may be related to variations in pain mechanisms across chronic pain conditions. Further evidence is needed to establish the robustness of these findings.

Favorable trends in these outcomes were also observed with tDCS combined with ET, although the effects did not reach statistical significance. tDCS can modulate cortical excitability and promote neuroplasticity, which may attenuate pain hypersensitivity through the regulation of pain-related neural circuits, thalamocortical pathways, and descending pain modulatory networks (Lefaucheur et al., 2017; Knotkova et al., 2013; Meeker et al., 2019). Its effects on motor cortex excitability and motor network connectivity may also facilitate motor learning and augment the effects of ET on functional recovery (Qi et al., 2024). However, these neuromodulatory effects are parameter-dependent, and therapeutic efficacy may be influenced by stimulation targets, intensity, and duration (Lefaucheur et al., 2017; Brunoni et al., 2012). In the present review, tDCS studies varied across several of these intervention characteristics, which may have influenced the observed treatment effects and should be considered when interpreting the findings. Although subgroup analyses showed greater effect estimates with rTMS combined with ET for several WOMAC outcomes, the three-level moderator analysis did not detect significant differences in treatment effects across NIBS modalities.

Finally, based on the limited available data, our study showed that NIBS combined with ET was associated with a favorable effect on gait speed, whereas no significant improvement in QoL was observed. A recent network meta-analysis suggested that certain exercise modalities may improve gait performance in patients with KOA, with the observed benefits varying across exercise types and follow-up periods (Yan et al., 2025). However, another randomized sham-controlled trial found no significant difference in gait speed between aquatic therapy combined with real and sham tDCS under either normal or dual-task conditions, although additional improvements in proprioception were observed with real tDCS (Kalkhoran and Khanmohammadi, 2025). Regarding QoL, a review has indicated that the level of evidence for the improvement of QoL in KOA patients through exercise is low to moderate (Lawford et al., 2024). Another systematic review has suggested that exercise may slightly enhance QoL immediately after treatment (Fransen et al., 2015). As a multidimensional subjective measure, QoL may be influenced by multiple factors, including psychological expectations, environmental adaptation, and variations in assessment tools. Although some studies have supported the beneficial effects of exercise on gait performance and QoL, evidence regarding NIBS combined with ET remains limited. In the present meta-analysis, each of these two secondary outcomes was assessed based on only three studies. Therefore, the effects of the combined intervention on gait speed and QoL require further investigation.

The findings of this study should be interpreted with full consideration of the limitations. First, clinical heterogeneity exists among the included studies, including differences in stimulation modality, stimulation target, intensity, treatment duration, and ET protocols. Although subgroup analyses were conducted to explore potential sources of heterogeneity, substantial heterogeneity remained in some subgroup analyses, which may affect the stability of the pooled estimates. There was also a marked imbalance in the number of studies across different NIBS modalities, with only two rTMS studies. These findings should therefore be regarded as exploratory, and no definitive conclusions can be drawn regarding differences in efficacy between NIBS techniques. Besides, most included studies had small sample sizes and insufficient long-term follow-up, which reduced the statistical power of the findings and limited the assessment of the duration of efficacy. The limited number of studies available also limited the reliability of publication bias assessment. Although three-level meta-analyses with RVE were used to accounted for within-study dependence in the VAS/NRS pain and WOMAC analyses, these models included only five to seven independent studies. This limited the confidence in the resulting estimates and the ability of moderator analyses to detect differences in treatment effects according to assessment time point, stimulation site, WOMAC subscale, or NIBS modality. Some studies also included samples with limited representativeness, such as studies enrolling patients of a single sex, which may affect the generalizability of results and the interpretation of mechanisms. Furthermore, the outcome measures are primarily subjective pain scales, lacking objective physical function tests and neurophysiological indicators, limiting the in-depth understanding of intervention effects and underlying mechanisms. Finally, current research on NIBS combined with exercise in KOA remains limited, with a predominant focus on tDCS-based interventions and insufficient evidence regarding other neuromodulation modalities, direct comparisons across stimulation sites, and alternative strategies targeting functional connectivity between brain regions. These limitations restrict the evaluation and comparison of different neuromodulation approaches and the identification of optimal intervention strategies.

To address these limitations, future studies should further standardize NIBS stimulation parameters and ET protocols, and direct comparative studies of different NIBS techniques combined with ET should be conducted to improve the reliability of efficacy comparisons across stimulation strategies and explore optimal combined intervention approaches. Future large-scale, long-term multicenter RCTs should be conducted to enhance statistical power and accuracy in evaluating long-term efficacy. The sample diversity should also be increased to enhance the generalizability of findings and the ability to elucidate mechanisms. To comprehensively quantify treatment efficacy, objective physical function tests and neurophysiological indicators should be integrated. Research on other NIBS approaches combined with exercise, such as theta-burst stimulation (Suppa et al., 2016) and focused ultrasound stimulation (Fomenko et al., 2018), should be expanded. In addition, direct comparisons across stimulation sites, as well as alternative strategies targeting inter-regional functional connectivity, such as cortico-cortical paired associative stimulation (ccPAS) (Di Luzio et al., 2024) and dual-site tACS (Saturnino et al., 2017), should be further explored. These efforts may help expand the evidence base and clarify the comparative effects of different neuromodulation strategies in KOA.

## 5 Conclusion

This study suggests that NIBS combined with ET was associated with significant reductions in the WOMAC total score, although the interpretation of the WOMAC findings was affected after accounting for within-study correlations. Subgroup analyses further suggest beneficial effects on the WOMAC total score at ≥ 12 weeks and with rTMS combined with ET. However, no significant improvements were observed in the pain measured by NRS or VAS. For gait speed and QoL, the limited evidence suggested a statistically significant improvement in gait speed, while no significant improvement in QoL was observed. Future high-quality clinical studies are needed to further validate the efficacy of the combined intervention and explore personalized treatment strategies to optimize the comprehensive management of KOA.

## Data Availability

The original contributions presented in the study are included in the article/Supplementary material, further inquiries can be directed to the corresponding author/s.
