# Supplementary material for: Effects of non-invasive brain stimulation combined with exercise training on pain and physical function in knee osteoarthritis: a systematic review and meta-analysis
Source: Front Hum Neurosci. 2026 Oct 28;20:1902298. doi: 10.3389/fnhum.2026.1902298 (PMC13751418; doi:10.3389/fnhum.2026.1902298)
Supplement: Supplementary file 1 [file Supplementary_file_1.docx]

**Supplementary documents**

Figure S1 Risk of bias assessment of included randomized controlled trials using the Cochrane Risk of Bias 2 (RoB 2) tool

Figure S2 Forest plot of subgroup analysis by time for the efficacy of non-invasive brain stimulation combined with exercise in reducing pain scores

Figure S3 Forest plot of subgroup analysis by stimulation site for the efficacy of non-invasive brain stimulation combined with exercise in reducing pain scores

Figure S4 Forest plot of subgroup analysis by stimulation modality for the efficacy of non-invasive brain stimulation combined with exercise in reducing pain scores

Figure S5 Forest plot for effects of non-invasive brain stimulation combined with exercise on the score of WOMAC pain subscale

Figure S6 Forest plot for effects of non-invasive brain stimulation combined with exercise on the score of WOMAC physical function subscale

Figure S7 Forest plot for effects of non-invasive brain stimulation combined with exercise on the score of WOMAC stiffness subscale

Figure S8 Forest plot of subgroup analysis by stimulation modality for the efficacy of non-invasive brain stimulation combined with exercise in improving WOMAC total score

Figure S9 Forest plot of subgroup analysis by stimulation modality for the efficacy of non-invasive brain stimulation combined with exercise on the score of WOMAC pain subscale

Figure S10 Forest plot of subgroup analysis by stimulation modality for the efficacy of non-invasive brain stimulation combined with exercise on the score of WOMAC function subscale

Figure S11 Forest plot for effects of non-invasive brain stimulation combined with exercise on gait speed score

Figure S12 Forest plot for effects of non-invasive brain stimulation combined with exercise on quality of life score

Figure S13 Sensitivity analysis for pain score

Figure S14 Forest plot for effects of non-invasive brain stimulation combined with exercise on pain score after exclusion of one study

Figure S15 Sensitivity analysis for WOMAC total score

Figure S16 Funnel plot for publication bias in studies reporting pain scores

Figure S17 Funnel plot for publication bias in studies reporting WOMAC total scores

Figure S18 Trim-and-fill funnel plot for pain scores

Figure S19 Trim-and-fill funnel plot for WOMAC total scores

Table S1 A record of search results

| Pubmed | | |
| --- | --- | --- |
| # | Query | Results |
| 1 | Osteoarthritis, Knee [MeSH Terms] | 32,059 |
| 2 | "Osteoarthritis of Knee"[Title/Abstract] OR "knee osteoarthrosis"[Title/Abstract] OR "Knee Osteoarthritis"[Title/Abstract] OR "Knee Osteoarthritides"[Title/Abstract] OR "knee arthrosis"[Title/Abstract] OR "gonarthrosis"[Title/Abstract] OR "femorotibial arthrosis"[Title/Abstract] | 22,738 |
| 3 | Transcranial Magnetic Stimulation [MeSH Terms] | 17,437 |
| 4 | Transcranial Direct Current Stimulation [MeSH Terms] | 6,498 |
| 5 | "Transcranial Magnetic Stimulation"[Title/Abstract] OR "Transcranial Random Noise Stimulation"[Title/Abstract] OR "Transcranial Electrical Stimulation"[Title/Abstract] OR "Transcranial Direct Current Stimulation"[Title/Abstract] OR "Transcranial Alternating Current Stimulation"[Title/Abstract] OR "tDCS"[Title/Abstract] OR "transcranial focused ultrasound stimulation"[Title/Abstract] OR "tFUS"[Title/Abstract] OR "Theta-burst stimulation"[Title/Abstract] OR "TBS"[Title/Abstract] OR "NIBS" [Title/Abstract] OR "brain stimulation"[Title/Abstract] | 59,071 |
| 6 | Exercise [MeSH Terms] | 278,648 |
| 7 | Motion [MeSH Terms] | 74,866 |
| 8 | Sports [MeSH Terms] | 236,782 |
| 9 | "physical workout"[Title/Abstract] OR "Physical Activity"[Title/Abstract] OR "fitness workout"[Title/Abstract] OR "fitness training"[Title/Abstract] OR "exertion"[Title/Abstract] OR "Exercise"[Title/Abstract] OR "effort"[Title/Abstract] OR "movement"[Title/Abstract] OR "motion"[Title/Abstract] OR "sport"[Title/Abstract] OR "competitive gymnastics"[Title/Abstract] OR "Athletic"[Title/Abstract] OR "training"[Title/Abstract] OR "detraining"[Title/Abstract] | 1,836,705 |
| 10 | (#1 OR #2) AND ((#3 OR #4 OR #5) AND (#6 OR #7 OR #8 OR #9)) | 27 |

| Embase | | |
| --- | --- | --- |
| # | Query | Results |
| #1 | 'knee osteoarthritis'/exp | 57454 |
| #2 | 'osteoarthritis of knee':ti,ab,kw OR 'knee osteoarthrosis':ti,ab,kw OR 'knee osteoarthritis':ti,ab,kw OR 'knee osteoarthritides':ti,ab,kw OR 'knee arthrosis':ti,ab,kw OR 'gonarthrosis':ti,ab,kw OR 'femorotibial arthrosis':ti,ab,kw | 36794 |
| #3 | 'transcranial magnetic stimulation'/exp | 41515 |
| #4 | 'transcranial direct current stimulation'/exp | 16474 |
| #5 | 'transcranial magnetic stimulation':ti,ab,kw OR 'transcranial random noise stimulation':ti,ab,kw OR 'transcranial electrical stimulation':ti,ab,kw OR 'transcranial direct current stimulation':ti,ab,kw OR 'transcranial alternating current stimulation':ti,ab,kw OR 'tdcs':ti,ab,kw OR 'transcranial focused ultrasound stimulation':ti,ab,kw OR 'tfus':ti,ab,kw OR 'theta-burst stimulation':ti,ab,kw OR 'tbs':ti,ab,kw OR 'nibs':ti,ab,kw OR 'brain stimulation':ti,ab,kw | 93548 |
| #6 | 'exercise'/exp | 554938 |
| #7 | 'motion'/exp | 238884 |
| #8 | 'sport'/exp | 255970 |
| #9 | 'training'/exp | 157480 |
| #10 | 'physical workout':ti,ab,kw OR 'physical activity':ti,ab,kw OR 'fitness workout':ti,ab,kw OR 'fitness training':ti,ab,kw OR 'exertion':ti,ab,kw OR 'exercise':ti,ab,kw OR 'effort':ti,ab,kw OR 'movement':ti,ab,kw OR 'motion':ti,ab,kw OR 'sport':ti,ab,kw OR 'competitive gymnastics':ti,ab,kw OR 'athletic':ti,ab,kw OR 'training':ti,ab,kw OR 'detraining':ti,ab,kw | 2443480 |
| #11 | (#1 OR #2) AND (#3 OR #4 OR #5) AND (#6 OR #7 OR #8 OR #9 OR #10) | 48 |

| Cochrane Library | | |
| --- | --- | --- |
| # | Query | Results |
| #1 | MeSH descriptor: [Osteoarthritis, Knee] explode all trees | 7300 |
| #2 | ('Osteoarthritis of Knee' OR 'knee osteoarthrosis' OR 'Knee Osteoarthritis' OR 'Knee Osteoarthritides' OR 'knee arthrosis' OR 'gonarthrosis' OR 'femorotibial arthrosis'):ti,ab,kw | 20276 |
| #3 | MeSH descriptor: [Transcranial Magnetic Stimulation] explode all trees | 2930 |
| #4 | MeSH descriptor: [Transcranial Direct Current Stimulation] explode all trees | 2301 |
| #5 | ('Transcranial Magnetic Stimulation' OR 'Transcranial Random Noise Stimulation' OR 'Transcranial Electrical Stimulation' OR 'Transcranial Direct Current Stimulation' OR 'Transcranial Alternating Current Stimulation' OR 'tDCS' OR 'transcranial focused ultrasound stimulation' OR 'tFUS' OR 'Theta-burst stimulation' OR 'TBS' OR 'NIBS' OR 'brain stimulation'):ti,ab,kw | 25599 |
| #6 | MeSH descriptor: [Exercise] explode all trees | 41320 |
| #7 | MeSH descriptor: [Motion] explode all trees | 2135 |
| #8 | MeSH descriptor: [Sports] explode all trees | 23243 |
| #9 | ('physical workout' OR 'Physical Activity' OR 'fitness workout' OR 'fitness training' OR 'exertion' OR 'Exercise' OR 'effort' OR 'movement' OR 'motion' OR 'sport' OR 'competitive gymnastics' OR 'Athletic' OR 'training' OR 'detraining'):ti,ab,kw | 335798 |
| #10 | (#1 OR #2) AND ((#3 OR #4 OR #5) AND (#6 OR #7 OR #8 OR #9)) | 71 |

| Web of Science | |
| --- | --- |
| Query | Results |
| "Osteoarthritis of Knee" OR "knee osteoarthrosis" OR "Knee Osteoarthritis" OR "Knee Osteoarthritides" OR "knee arthrosis" OR "gonarthrosis" OR "femorotibial arthrosis" (Topic) | 38536 |
| "Transcranial Magnetic Stimulation" OR "Transcranial Random Noise Stimulation" OR "Transcranial Electrical Stimulation" OR "Transcranial Direct Current Stimulation" OR "Transcranial Alternating Current Stimulation" OR "tDCS" OR "transcranial focused ultrasound stimulation" OR "tFUS" OR "Theta-burst stimulation" OR "TBS" OR "NIBS" OR "brain stimulation" (Topic) | 87435 |
| "physical workout" OR "Physical Activity" OR "fitness workout" OR "fitness training" OR "exertion" OR "Exercise" OR "effort" OR "movement" OR "motion" OR "sport" OR "competitive gymnastics" OR "Athletic" OR "training" OR "detraining" (Topic) | 4094695 |
| #2 AND #3 | 16119 |
| #1 AND #4 | 36 |

Table S2 Eligibility criteria of the included studies

| References | Diagnostic criteria | Key inclusion criteria | Key exclusion criteria |
| --- | --- | --- | --- |
| Wang et al. (2025) | ACR criteria; KL grade II–III | Age 50–75 years; KOA duration >3 months (non-acute phase); VAS ≤5/10 | Previous knee trauma or surgery; rheumatoid arthritis or other joint diseases; patients with severe organ failure, severe cardiovascular and cerebrovascular diseases and other contraindications to  exercise; malignant tumors |
| Kalkhoran and Khanmohammadi (2025) | Clinical diagnosis based on physical examination and radiography; KL grade ≥I | Women aged 63–68 years; physically inactive (<1 h/week of physical activity); hemodynamically stable (BP <140/90 mmHg); BMI <40 kg/m² | Previous knee surgery or gait-affecting joint disease; untreated thyroid disease; severe respiratory, neurological or musculoskeletal disorders; recent corticosteroid injection; contraindications to tDCS; left ventricular dysfunction; acute infection or illness |
| Kaboodi et al. (2025) | ACR criteria; KL grade ≥II | Age 50–70 years; chronic knee pain (≥3 months); VAS ≥4/10; stable pain medication regimen | Previous knee ligament/meniscus injury or surgery; concomitant hip OA or systemic rheumatic disease; recent physiotherapy, intra-articular injection, or exercise (<3 months); cardiovascular or neuromuscular disorders; cognitive impairment or severe depression; BMI >40 kg/m²; contraindications to tDCS; awaiting arthroplasty |
| Chang et al. (2025) | ACR criteria | Age ≥50 years; knee pain ≥3 months; average NRS ≥4/10 during the previous week | Previous knee replacement or high tibial osteotomy; knee surgery or joint injection (<6 months); systemic arthritis; previous knee fracture or malignancy; lower-limb disorders affecting function; neurological or psychiatric disorders; use of neuroactive drugs; recent knee strengthening exercise (<6 months); contraindications to rTMS; resting motor threshold >80%; planned knee surgery |
| Boonprasit et al. (2025) | ACR criteria; KL grade I–III | Age 50–90 years; knee pain >3 months; VAS 40–70/100 mm; able to sit for ≥20 min | Previous knee replacement or acute knee inflammation; recent NSAID use (<2 weeks) or intra-articular injection (steroid <3 months; hyaluronic acid <6 months); hemodynamic or neurological instability; major depressive disorder; previous NIBS; contraindications to tDCS |
| Montilla-Herrador et al. (2024) | OARSI criteria; KL grade I–IV | Age >50 years; chronic pain >6 months; VAS >4/10 | Contralateral knee replacement; invasive pain intervention (<6 weeks); acute musculoskeletal disorders; severe heart disease or comorbidities limiting physical activity; severe sensory impairment; cognitive impairment based on MMSE assessment; BMI >45 kg/m²; neuropsychiatric disorders or related medication; metallic implants |
| Chang et al. (2017) | ACR criteria | Age >50 years; knee pain; VAS ≥40/100 mm during walking in the previous week | Detailed exclusion criteria were not reported in the published article; TMS safety-related exclusions included epilepsy, brain lesions or infarcts, neurological disorders, cognitive impairment, pregnancy, and implanted metallic or orthopedic devices |
| Rahimi et al. (2021) | ACR criteria; KL grade II–III | Age 50–65 years; right-leg dominance (or dominant side in bilateral KOA); VAS ≥4/10; knee range of motion limitation | Traumatic knee injury; severe joint inflammation; secondary KOA due to rheumatological disease; neurological disorders affecting limb function; epilepsy or mental disorders; advanced cardiovascular disease; inability to walk independently; previous knee surgery (<6 months); recent KOA treatment or rehabilitation |

Notes: ACR = American College of Rheumatology; OARSI = Osteoarthritis Research Society International; KL = Kellgren–Lawrence grading system; VAS = Visual Analog Scale; NRS = Numeric Rating Scale; BMI = Body Mass Index; BP = Blood Pressure; KOA = Knee Osteoarthritis; OA = Osteoarthritis; NIBS = Non-invasive Brain Stimulation; tDCS = transcranial direct current stimulation; rTMS = repetitive Transcranial Magnetic Stimulation; NSAIDs = Non-steroidal Anti-inflammatory Drugs; MMSE = Mini-Mental State Examination; The reference numbers correspond to the citation numbers in the main manuscript; Eligibility criteria were extracted from the published articles and their supplementary materials, where available.

**References**

Wang, Y., Feng, W., Yang, K., Ding, Y., Ma, B., and Jiang, L. (2025). Effectiveness of high-frequency repetitive transcranial magnetic stimulation combined with quadriceps strength training in the treatment of knee osteoarthritis: a randomized controlled trial. Ther. Adv. Musculoskelet Dis. 17:1759720x251370979. doi: 10.1177/1759720x251370979

Kalkhoran, Z. B., and Khanmohammadi, R. (2025). The effects of aquatic therapy combined with transcranial direct current stimulation (tDCS) on proprioception and gait speed in older adults with knee osteoarthritis: an eight-week randomized sham-controlled trial. BMC Geriatr. 25:676. doi: 10.1186/s12877-025-06253-5

Kaboodi, A. T., Gandomi, F., Soufivand, P., and Assar, S. (2025). The impacts of transcranial direct current stimulation combined with aquatic neuromuscular training on pain, function, kinesiophobia, knee instability, and quality of life in knee osteoarthritis: a double-blind randomized trial. J. Neuroeng. Rehabil. 22:151. doi: 10.1186/s12984-025-01670-4

Chang, W. J., Chiang, A., Chowdhury, N., Adie, S., Naylor, J. M., Finn, H., et al. (2025). Repetitive transcranial magnetic stimulation as an adjunct to quadriceps strengthening exercise in knee osteoarthritis: a pilot randomised controlled trial. BMJ Open 15:e097293. doi: 10.1136/bmjopen-2024-097293

Boonprasit, K., Stonsaovapak, C., and Piravej, K. (2025). Effect of transcranial direct current stimulation at the left dorsolateral prefrontal cortex combined with quadriceps strengthening exercise in chronic knee osteoarthritis: a double-blind, randomized, sham-controlled trial. Am. J. Phys. Med. Rehabil. 105, 200–207. doi: 10.1097/phm.0000000000002852

Montilla-Herrador, J., Lozano-Meca, J., Lozano-Guadalajara, J. V., and Gacto-Sánchez, M. (2024). The efficacy of the addition of tDCS and TENS to an education and exercise program in subjects with knee osteoarthritis: a randomized controlled trial. Biomedicines 12:1186. doi: 10.3390/biomedicines12061186

Chang, W. J., Bennell, K. L., Hodges, P. W., Hinman, R. S., Young, C. L., Buscemi, V., et al. (2017). Addition of transcranial direct current stimulation to quadriceps strengthening exercise in knee osteoarthritis: a pilot randomised controlled trial. PLoS One 12:e0180328. doi: 10.1371/journal.pone.0180328

Rahimi, F., Nejati, V., Nassadj, G., Ziaei, B., and Mohammadi, H. K. (2021). The effect of transcranial direct stimulation as an add-on treatment to conventional physical therapy on pain intensity and functional ability in individuals with knee osteoarthritis: a randomized controlled trial. Neurophysiol. Clin. 51, 507–516. doi: 10.1016/j.neucli.2021.06.002

Table S3 Certainty of evidence assessed using the GRADE approach for each outcome

| **Certainty assessment** | | | | | | | **№ of patients** | | **Effect** | **Certainty** |
| --- | --- | --- | --- | --- | --- | --- | --- | --- | --- | --- |
| **№ of studies** | **Study design** | **Risk of bias** | **Inconsistency** | **Indirectness** | **Imprecision** | **Other considerations** | **[intervention]** | **[comparison]** |  |  |
| **Pain (follow-up: mean 6.3 weeks; assessed with: VAS/NRS)** | | | | | | | | | | |
| 7 | randomised trials | not serious | serious^a^ | not serious | serious^b^ | none | 162 | 122 | SMD **0.41 SD lower** (0.83 lower to 0.01 highter) | ⨁⨁◯◯ Low^a,b^ |
| **Physical Function (follow-up: mean 6.7 weeks; assessed with: WOMAC; Scale from: 0 to 96)** | | | | | | | | | | |
| 6 | randomised trials | not serious | not serious | not serious | serious^c^ | none | 107 | 102 | MD 6.76 **lower** (11.85 lower to 1.68 lower) | ⨁⨁⨁◯ Moderate^c^ |
| **Gait speed (follow-up: mean 14.8 weeks; assessed with: 10-meter walk test)** | | | | | | | | | | |
| 3 | randomised trials | serious^d^ | not serious | not serious | serious^e^ | none | 97 | 57 | SMD **0.35 SD lower** (0.69 lower to 0.01 lower) | ⨁⨁◯◯ Low^d,e^ |
| **Quality of life (follow-up: mean 2.8 weeks; assessed with: OAKHQOL/EQ-5D/SF-36)** | | | | | | | | | | |
| 3 | randomised trials | serious^f^ | not serious | not serious | serious^g^ | none | 56 | 56 | SMD **0.20 SD higher** (0.17 lower to 0.57 higher) | ⨁⨁◯◯ Low^f,g^ |

**CI:** confidence interval; **MD:** mean difference; **SMD:** standardised mean difference

**Explanations**

a. Downgraded one level for serious inconsistency because substantial heterogeneity was observed (I² = 67.3%). Sensitivity analysis indicated that removal of Rahimi 2021 substantially reduced heterogeneity, suggesting variability in treatment effects across studies.

b. Downgraded one level for imprecision because the 95% confidence interval was wide, crossing the line of no effect and ranging from a large reduction in pain (SMD -0.83) to a trivial increase in pain (SMD 0.01), and the total sample size did not reach the optimal information size.

c. Downgraded one level for serious imprecision because the total sample size did not reach the optimal information size, resulting in uncertainty regarding the precision of the estimated treatment effect.

d. One of three included studies (30.2% weight) was at high risk of bias and contributed substantially to the pooled estimate.

e. Downgraded one level for imprecision because of the limited sample size and the wide confidence interval, indicating substantial uncertainty in the magnitude of the effect.

f. Downgraded one level for serious risk of bias because one of the three included studies (36.0% weight) was judged to be at high risk of bias.

g. Downgraded one level for serious imprecision because the 95% confidence interval crossed the line of no effect, and the total sample size was limited.

General note. Applicable to Tables S4–S7. k denotes the number of effect sizes. Effect estimates are expressed as Hedges’ g in Tables S4–S6 and as mean differences (MDs) in Table S7, with negative values indicating greater reductions in pain or WOMAC scores in the NIBS + ET group compared with the control group. Three-level meta-analyses were estimated using restricted maximum likelihood (REML), with effect sizes nested within studies. σ² Level 3 represents between-study variance, whereas σ² Level 2 represents variance attributable to differences among effect sizes within studies. ρ denotes the assumed within-study correlation used to construct the variance-covariance matrix; ρ = 0.7 was used for primary analyses, with ρ = 0.5 and 0.9 examined in sensitivity analyses. Robust variance estimation (RVE) was performed using the CR2 small-sample correction with Satterthwaite degrees of freedom.

Abbreviations: CI, confidence interval; df, degrees of freedom; DLPFC, dorsolateral prefrontal cortex; ET, exercise therapy; MD, mean difference; NIBS, non-invasive brain stimulation; NRS, Numeric Rating Scale; REML, restricted maximum likelihood; RVE, robust variance estimation; S1, primary somatosensory cortex; VAS, Visual Analog Scale; WOMAC, Western Ontario and McMaster Universities Osteoarthritis Index.

Table S4 Three-level meta-analysis, robust variance estimation, and moderator analysis of VAS/NRS pain estimates with within-study correlations across assessment time points

Panel A. Pooled effects from three-level meta-analysis and robust variance estimation

| Analysis | k (effect sizes) | Studies | Assumed ρ | Three-level Hedges’ g (95% CI) | p | σ² (Level 3) | σ² (Level 2) | RVE Hedges’ g (95% CI) | RVE df | RVE p |
| --- | --- | --- | --- | --- | --- | --- | --- | --- | --- | --- |
| Primary analysis: VAS/NRS pain with multiple assessment time points | 10 | 7 | 0.7 | −0.40 (−0.88, 0.09) | 0.098 | 0.214 | 0.000 | −0.40 (−0.93, 0.13) | 5.94 | 0.117 |
| ρ sensitivity | 10 | 7 | 0.5 | −0.41 (−0.89, 0.08) | 0.091 | 0.217 | 0.000 | −0.41 (−0.94, 0.12) | 5.94 | 0.110 |
| ρ sensitivity | 10 | 7 | 0.9 | −0.39 (−0.88, 0.10) | 0.105 | 0.203 | 0.016 | −0.39 (−0.93, 0.14) | 5.94 | 0.123 |

Panel B. Moderator analysis within the three-level model

| Analysis | Moderator | Assumed ρ | Omnibus moderator test | p |
| --- | --- | --- | --- | --- |
| VAS/NRS pain with multiple assessment time points (k = 10; 7 studies) | Assessment time point | 0.7 | F(2, 7) = 0.10 | 0.907 |

Specific note. This analysis included multiple estimates from the same studies at different assessment time points; within-study correlations among these repeated estimates were therefore accounted for in the three-level models.

Table S5 Three-level meta-analysis, robust variance estimation, and moderator analysis of VAS/NRS pain estimates with within-study correlations across stimulation sites

Panel A. Pooled effects from three-level meta-analysis and robust variance estimation

| Analysis | k (effect sizes) | Studies | Assumed ρ | Three-level Hedges’ g (95% CI) | p | σ² (Level 3) | σ² (Level 2) | RVE Hedges’ g (95% CI) | RVE df | RVE p |
| --- | --- | --- | --- | --- | --- | --- | --- | --- | --- | --- |
| Primary analysis: stimulation-site-specific VAS/NRS pain estimates | 9 | 7 | 0.7 | −0.39 (−0.86, 0.07) | 0.086 | 0.129 | 0.043 | −0.39 (−0.89, 0.11) | 5.92 | 0.102 |
| ρ sensitivity | 9 | 7 | 0.5 | −0.39 (−0.86, 0.07) | 0.086 | 0.154 | 0.018 | −0.39 (−0.89, 0.11) | 5.92 | 0.102 |
| ρ sensitivity | 9 | 7 | 0.9 | −0.39 (−0.86, 0.07) | 0.086 | 0.104 | 0.067 | −0.39 (−0.89, 0.11) | 5.92 | 0.102 |

Panel B. Moderator analysis within the three-level model

| Analysis | Moderator | Assumed ρ | Omnibus moderator test | p |
| --- | --- | --- | --- | --- |
| Stimulation-site-specific VAS/NRS pain estimates (k = 9; 7 studies) | Stimulation site | 0.7 | F(2, 6) = 2.38 | 0.174 |

Specific note. This analysis included multiple stimulation-site-specific estimates derived from the same randomized trials; therefore, within-study dependence among these estimates was accounted for in the three-level models. The stimulation-site moderator analysis should be interpreted as exploratory because of the limited number of studies, particularly for DLPFC and S1.

Table S6 Three-level meta-analysis, robust variance estimation, and moderator analyses for correlated WOMAC outcomes

Panel A. Pooled effects from three-level meta-analysis and robust variance estimation

| Analysis | k (effect sizes) | Studies | Assumed ρ | Three-level Hedges’ g (95% CI) | p | σ² (Level 3) | σ² (Level 2) | RVE Hedges’ g (95% CI) | RVE df | RVE p |
| --- | --- | --- | --- | --- | --- | --- | --- | --- | --- | --- |
| **Primary analysis: WOMAC subscales** | 13 | 5 | **0.7** | **−0.38 (−0.81, 0.05)** | **0.081** | 0.091 | 0.008 | **−0.38 (−0.94, 0.18)** | 3.93 | **0.135** |
| ρ sensitivity | 13 | 5 | 0.5 | −0.38 (−0.82, 0.06) | 0.082 | 0.113 | 0.000 | −0.38 (−0.95, 0.19) | 3.94 | 0.134 |
| ρ sensitivity | 13 | 5 | 0.9 | −0.38 (−0.81, 0.06) | 0.082 | 0.069 | 0.032 | −0.38 (−0.95, 0.19) | 3.92 | 0.136 |
| **Sensitivity analysis: WOMAC total + subscales** | 19 | 6 | **0.7** | **−0.43 (−0.82, −0.05)** | **0.030** | 0.095 | 0.015 | **−0.43 (−0.91, 0.05)** | 4.94 | **0.068** |
| ρ sensitivity | 19 | 6 | 0.5 | −0.44 (−0.83, −0.05) | 0.031 | 0.122 | 0.000 | −0.44 (−0.92, 0.05) | 4.95 | 0.069 |
| ρ sensitivity | 19 | 6 | 0.9 | −0.43 (−0.81, −0.05) | 0.030 | 0.070 | 0.037 | −0.43 (−0.91, 0.05) | 4.92 | 0.068 |

Panel B. Moderator analyses within the three-level models

| Analysis | Moderator | Assumed ρ | Omnibus moderator test | p |
| --- | --- | --- | --- | --- |
| Primary: WOMAC subscales (k = 13; 5 studies) | WOMAC domain | 0.7 | F(2, 10) = 0.17 | 0.843 |
| Primary: WOMAC subscales (k = 13; 5 studies) | NIBS modality | 0.7 | F(1, 11) = 2.85 | 0.119 |
| Sensitivity: WOMAC total + subscales (k = 19; 6 studies) | WOMAC domain | 0.7 | F(3, 15) = 0.52 | 0.676 |
| Sensitivity: WOMAC total + subscales (k = 19; 6 studies) | NIBS modality | 0.7 | F(1, 17) = 1.58 | 0.226 |

Specific note. The primary analysis included WOMAC pain, stiffness, and physical function subscales; WOMAC total score was additionally included in the sensitivity analysis. Moderator analyses were considered exploratory given the limited number of independent studies.

Table S7 Three-level meta-analysis, robust variance estimation, and moderator analysis for correlated WOMAC total score estimates across assessment time points

Panel A. Pooled effects from three-level meta-analysis and robust variance estimation

| **Analysis** | **k (effect sizes)** | **Studies** | **Assumed ρ** | **Three-level MD (95% CI)** | **p** | **σ² (Level 3)** | **σ² (Level 2)** | **RVE MD (95% CI)** | **RVE df** | **RVE p** |
| --- | --- | --- | --- | --- | --- | --- | --- | --- | --- | --- |
| **Primary analysis: WOMAC total score with multiple assessment time points** | **9** | **6** | **0.7** | **−6.49 (−12.65, −0.33)** | **0.041** | **22.014** | **0.000** | **−6.49 (−13.63, 0.65)** | **4.57** | **0.066** |
| **ρ sensitivity** | **9** | **6** | **0.5** | **−6.61 (−12.69, −0.52)** | **0.037** | **21.818** | **0.000** | **−6.61 (−13.66, 0.44)** | **4.60** | **0.061** |
| **ρ sensitivity** | **9** | **6** | **0.9** | **−6.08 (−12.48, 0.33)** | **0.060** | **23.228** | **1.996** | **−6.08 (−13.43, 1.27)** | **4.61** | **0.086** |

Panel B. Moderator analysis within the three-level model

| **Analysis** | **Moderator** | **Assumed ρ** | **Omnibus moderator test** | **p** |
| --- | --- | --- | --- | --- |
| **WOMAC total score with multiple assessment time points (k = 9; 6 studies)** | **Assessment time point** | **0.7** | **F(2, 6) = 2.96** | **0.127** |

Specific note. This analysis included multiple WOMAC total score estimates derived from the same studies at different assessment time points; therefore, within-study correlations among repeated estimates were accounted for in the three-level models.
